# Extending Whole Slide Imaging: Color Darkfield Internal Reflection Illumination (DIRI) for Biological Applications

**DOI:** 10.1371/journal.pone.0167774

**Published:** 2017-01-13

**Authors:** Yoshihiro Kawano, Kana Namiki, Atsushi Miyawaki, Takuji Ishikawa

**Affiliations:** 1 The Department of Biomedical Engineering, Graduate School of Biomedical Engineering, Tohoku University, Sendai, Miyagi, Japan; 2 Olympus Corporation, Shinjuku-Ku, Tokyo, Japan; 3 Cell Function & Dynamics, Brain Science Institute, RIKEN, Wako, Saitama, Japan; 4 Department of Finemechanics, Tohoku University, Sendai, Miyagi, Japan; City University London, UNITED KINGDOM

## Abstract

Whole slide imaging (WSI) is a useful tool for multi-modal imaging, and in our work, we have often combined WSI with darkfield microscopy. However, traditional darkfield microscopy cannot use a single condenser to support high- and low-numerical-aperture objectives, which limits the modality of WSI. To overcome this limitation, we previously developed a darkfield internal reflection illumination (DIRI) microscope using white light-emitting diodes (LEDs). Although the developed DIRI is useful for biological applications, substantial problems remain to be resolved. In this study, we propose a novel illumination technique called color DIRI. The use of three-color LEDs dramatically improves the capability of the system, such that color DIRI (1) enables optimization of the illumination color; (2) can be combined with an oil objective lens; (3) can produce fluorescence excitation illumination; (4) can adjust the wavelength of light to avoid cell damage or reactions; and (5) can be used as a photostimulator. These results clearly illustrate that the proposed color DIRI can significantly extend WSI modalities for biological applications.

## Introduction

The complexity of the human body makes it difficult to understand. For example, it consists of between 50 and 75 trillion cells, and its genes are capable of creating over 20,000 proteins. Optical microscopy is the main tool used to investigate the structure and functions of cells, tissues, and organs in the human body. Scientists have a wide variety of imaging techniques and tools at their disposal to better understand complicated biological environments. Whole slide imaging (WSI) or so call vertual slide technology is one of the core imaging techniques, which enables automated imaging and allows scientists to observe large samples. Microscopes performing WSI can easily be connected to the Internet, raising the prospect of Internet of Things applications. The observation area of WSI is not limited to the field of view of an objective lens [1–5].

**Fig 1:**
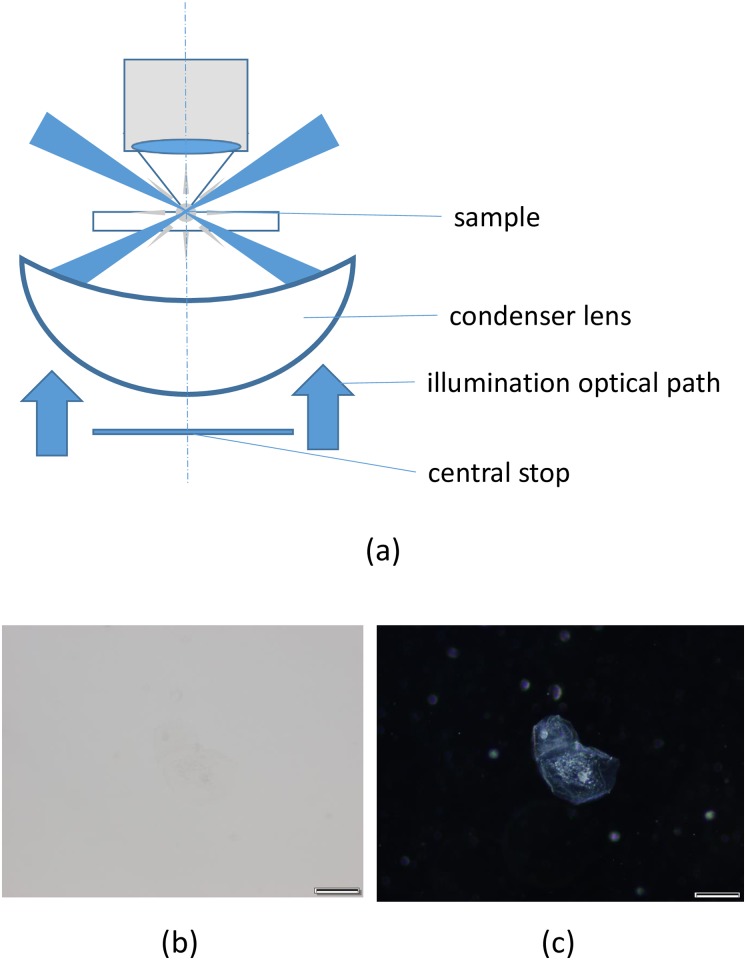
Cheek cell image using brightfield and darkfield microscopy. (a) Schematic diagrams of a darkfield condenser, (b) brightfield microscopy image, and (c) darkfield microscopy image of a cheek cell. Scale bars in the Figs represent 5 μm. Images were taken with UplanSapo 60× oil (NA 1.35). All images (b, c) were taken using the color camera.

Darkfield microscopy combined with WSI is often used to observe the details of tissue and cell structures. The main advantage of darkfield microscopy is that unstained samples can be observed due to a mismatch in the refractive index, which generates image contrast, although staining with an appropriate color is often required [6]. The typical set-up of transmitted darkfield illumination consists of a halogen burner bulb with either a central stop for darkfield in a standard brightfield condenser or a dedicated substage darkfield condenser. Central axial illumination from the halogen burner bulb is blocked by the darkfield central stop. Peripheral illumination obliquely illuminates a specimen contained on a slide from the bottom. Thus, only forward-scattered light or refracted light from the specimen enters the objective lens (Fig 1a), which creates darkfield images. We show the advantages of darkfield microscopy using real samples. Fig 1b shows a brightfield image of cheek cells using UPlanSApo 60× oil, numerical aperture (NA) 1.35 (Olympus, Tokyo, Japan), with an extended magnification lens of 2.5×. The human cheek cell (epithelial cell) sample was freshly taken using Q-chip and diluted in phosphate-buffered solution (PBS). The image contrast was low, making it difficult to observe the cells. A darkfield image of the cheek cells is shown in Fig 1c; in this image, the background is very dark, which results in the granules inside the cells being clearly resolved.

Conventional darkfield units are commercially available for a standard microscope. There are two types of darkfield condensers: dry and oil. A dry darkfield condenser cannot be used with objective lenses that have a NA > 0.8, because the illumination cone passes directly through the objective lens and eliminates the dark background of the image. An oil darkfield condenser, on the other hand, can accept such a high-NA lens. However, if a user needs to switch from an oil lens to a dry lens, the condenser also must be switched back to the dry darkfield condenser. This necessitates the disruptive and somewhat laborious process of removing the oil from the slide, which is incompatible with automated WSI.

To overcome this problem, we previously developed darkfield internal reflection illumination (DIRI) with white light-emitting diodes (LEDs) [7, 8]. DIRI eliminates sources of light from the top and bottom of a sample using a side-illuminated darkfield, which is an improved version of a Hausmann’s darkfield illuminator [9], occasionally used for brain imaging [10–13]. Although the developed DIRI is useful [7, 8], it has substantial limitations that must be addressed to make it more useful for biological applications. For example, the original DIRI cannot control the wavelength of light, which is essential for fluorescence excitation. Controlling the wavelength is also important for avoiding cell damage or reactions.

In this study, therefore, we developed a novel color DIRI system using three-color LEDs. This modification dramatically improves the capability of the system. Specifically, it enables users to control the wavelength of light, which is useful for fluorescence excitation, avoiding cell damage and reactions. We show that our color DIRI has a color balance function and is effective for samples with or without staining. Moreover, the system can be used to investigate phototactic activities of cells. We use *Euglena gracilis* as a model microorganism [14–19], and show that the color DIRI can actually induce phototactic behavior of cells. Lastly, we discuss the advantages of the color DIRI compared with Hausmann’s darkfield and white DIRI. Our ultimate goal is to develop an automated microscope system with multiple modes of use via color DIRI.

## Materials and Methods

### Sample preparation

#### TMA sample

We used an 11-core TMA sample (BSB0297; BioSB, Santa Barbara, CA, USA). The TMA consisted of 11 2-mm cores of formalin-fixed, paraffin-embedded tissues. The array configuration allows validation of reagents for immunohistochemistry applications. The 11-core TMA contains the following tissues: placenta, colon, prostate, skin, thyroid, liver, brain, kidney, tonsil, breast, and fallopian tube. We stained TMA samples using the primary antibody p40 for a brown color (DAB staining) and the primary antibody TTF-1 for magenta via a Polydetector Detection Systems staining kit (BioSB, Santa Barbara, CA, USA).

#### Brain slice sample

Wild-type mice (C57BL/6J, 12 weeks old) and yellow fluorescence protein (YFP-H) mice (23 weeks old) were used. The YFP-H mouse line was provided to Riken by Drs. J. R. Sanes and J. W. Lichtman. The YFP-H mouse lines were maintained by crossing with C57BL/6J mice for several generations. The experimental procedures and housing conditions for the animals were approved by the Riken Institute’s Animal Experiments Committee, and all animals were cared for and treated humanely in accordance with the Institutional Guidelines for Experiments Using Animals.

The mice were anesthetized using pentobarbital (Somnopentyl, 60 mg/kg body weight; Kyouritsu Seiyaku, Tokyo, Japan) and transcardially perfused with ice-cold PBS containing 10 U/mL heparin (Mochida Pharmaceutical Co. Ltd., Tokyo, Japan), followed by 4% (wt/vol) paraformaldehyde (PFA)/phosphate-buffered saline (PBS) (−). The dissected brains were subjected to post-fixation in 4% PFA/PBS (−) at 4°C overnight and embedded in Tissue-Tek (O.C.T. compound; Sakura, Torrance, CA, USA). A YFP-H mouse brain was cut into 50-μm-thick sagittal sections with a cryostat (CM1860; Leica, Wetzlar, Germany).

Before diaminobenzidine (DAB) staining, 30-μm-thick sections of wild-type mouse produced using a cryostat were treated with 3% H_2_O_2_ in MeOH for 30 min. The brain sections were permeabilized with 0.1% Triton X-100 in PBS (−) for 5 min and then treated with 1% BSA in PBS for 30 min. The sections were incubated with primary antibody [anti-NeuN monoclonal antibody (Millipore #MAB377, 1:400)] in PBS-T overnight at 4°C, and incubated with secondary antibody [HRP-labeled anti-mouse IgG (MBL #PM009-7, 1:1000)] for 30 min at room temperature. Then, the sections were incubated with secondary antibody [HRP-labeled anti-mouse IgG (MBL #PM009-7)] for 30 min at room temperature. Finally, the sections were incubated in DAB substrate solution (#SK-4100; Vector Laboratories, Burlingame, CA, USA), in accordance with the manufacturer’s protocol.

#### *Euglena gracilis* sample

In this study, we used *Euglena gracilis* as a model microorganism to show that the color DIRI is useful for investigating the photo responses of cells. *E*. *gracilis* is a unicellular flagellate in fresh water. Photo responses of *E*. *gracilis* have been studied for over a century [14–19]. Blue and green light illumination of *E*. *gracilis* induces positive or negative phototactic responses, resulting in accumulation or avoidance, respectively [16, 17]. The phototactic responses of *E*. *gracilis* to light of different wavelengths ranging from near ultraviolet (UV) to red were reported previously [19]. *E*. *gracilis* was cultivated using diluted water with a plant nutrition solution (Pot in AO; Fumakilla Limited, Tokyo, Japan) in a bottle for 10 days. The sample cells were loaded between the coverslip and microscope slide glass. We introduced a spacer to create a gap. This gap between the slide glass and the coverslip was approximately 0.4 mm.

### Equipment set-up

#### Microscope set-up

**Fig 2:**
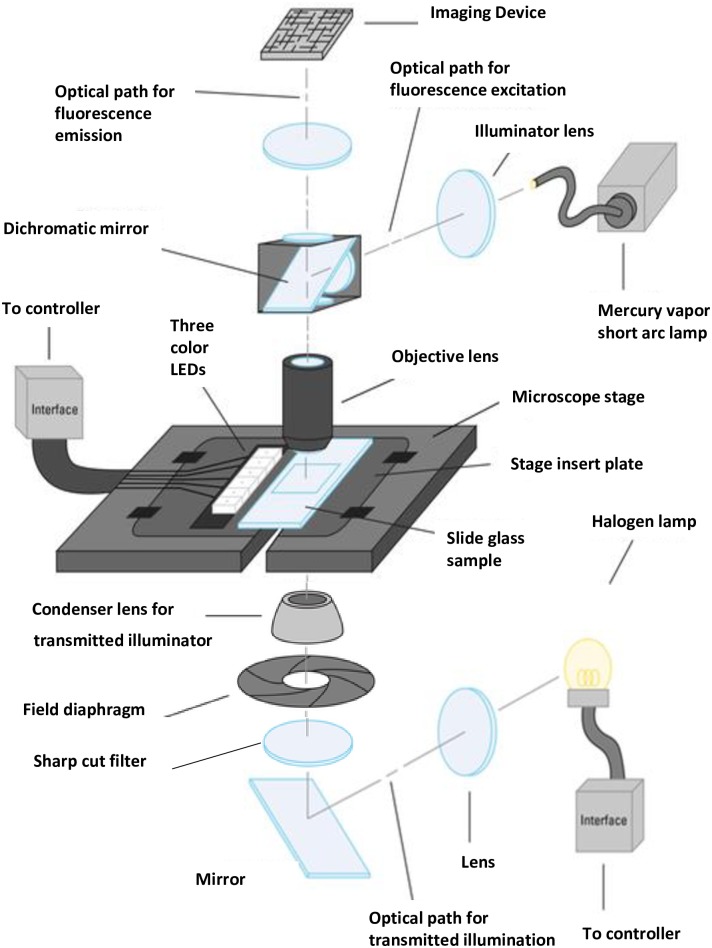
Schematic of the whole slide imaging (WSI) system with darkfield internal reflection illumination (DIRI). DIRI was incorporated into the WSI system’s motorized stage. Three color light-emitting diodes (LEDs) illuminate the slide glass from the side, and the specimen scatters this light. The scattered light is then incident on the objective lens above the stage. The dichromatic mirror on the motorized turret of the microscope can be removed from the light path when acquiring darkfield images. A tube lens above the dichromatic mirror focuses the sample image onto the imaging device. A charge-coupled device camera then captures the image. A sharp cut-off filter is placed between the field diaphragm and the mirror.

In all experiments, the base system comprised the Virtual Slide System VS120 (Olympus, Tokyo, Japan) and VS-ASW WSI software (ver. 2.8). A schematic diagram of the optical layout is shown in Fig 2. The WSI system set-up included a microscope imaging system with cameras, an objective lens, a motorized focus system, and a motorized X-Y stage. Two cameras were used: a black and white camera (Orca R2; Hamamatsu Photonics, Hamamatsu, Japan) and a color camera (VC50, CCD; Olympus Soft Imaging Solutions, Münster, Germany). This system contained a standard substage condenser, a transmitted brightfield illuminator, a fluorescence illuminator, and the color DIRI system. This configuration allowed automated acquisition of a specimen image. The acquisition parameters were configured using software to select the magnification (objective lens) and observation method (e.g. transmitted brightfield, fluorescence); the software controls the positioning of the stage and focus, such that images can be taken automatically. The color DIRI system was a prototype, and there was no commercial version. The physical arrangement of the LEDs and slide is the same with Fig 1 in our previous publication [7]. To avoid photo responses of *E*. *gracilis*, we added the sharp cut-off filter SC68 and reduced the exposure to visible light. This SC68 filter transmits light with a wavelength longer than 680 nm.

#### Color DIRI set-up

**Fig 3:**
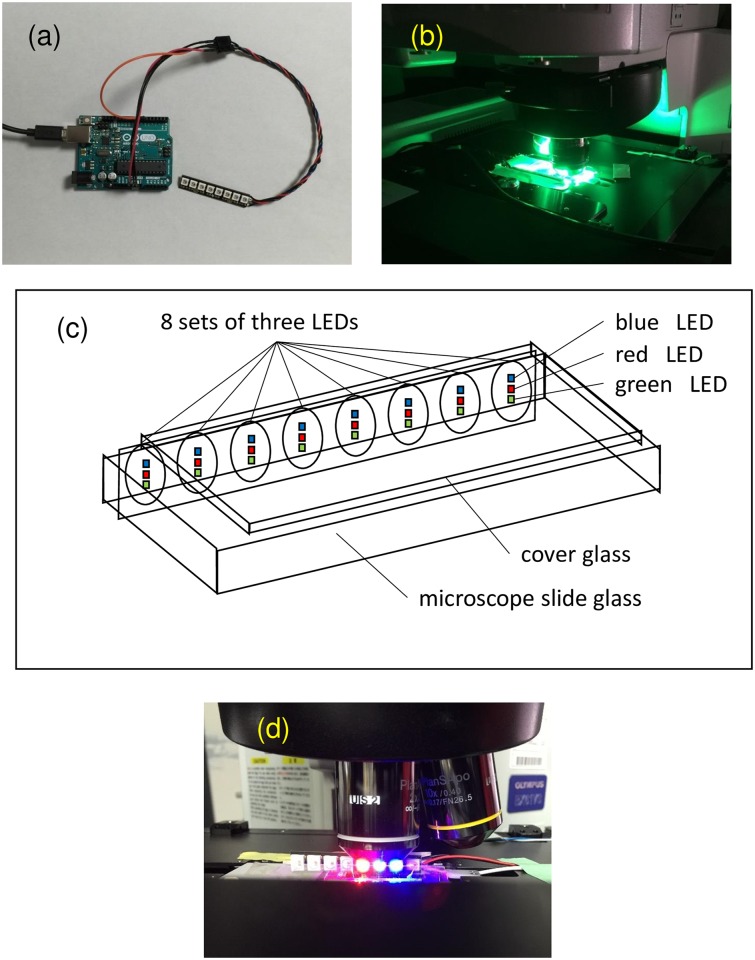
DIRI with three-color-LED array. (a) The three-color-LED array and controller, (b) a sample illuminated with green-LED array, (c) a schematic diagram of the three-color LEDs and a slide glass sample, and (d) a sample illuminated with red (left), green (middle), and blue (right) LEDs in the *E gracilis* experiment.

The previous method of white DIRI illumination [7, 8] uses a thin array of white LEDs. The present version of DIRI illumination uses a three-color-LED array (Fig 3), which is commercially available (NeoPixel Stick–ID 14268; Adafruit, New York, NY, USA). This LED array uses eight sets of three-color (red, blue, and green) LEDs (Worldsemi WS2812 5050 RGB). The dimensions are 51.1 × 10.22 × 3.19 mm. The WS2812 datasheet shows that the red, green, and blue LEDs emit light over the wavelength ranges of 620–630 nm (550–700 mcd), 515–530 nm (1100–1400 mcd), and 465–475 nm (200–400 mcd), respectively. The physical arrangement of the LEDs is shown in Fig 3(c). The distance between the blue and green LEDs is about 1.2 mm. The red LED is placed in between the blue and green LEDs.

Each LED is addressable by its internal driver chip. The operation frequency is 800 kHz. Each individual LED is controlled by an Arduino UNO (Arduino, Turin, Italy) microcontroller, with a clock speed of 16 MHz; the brightness of the LED light can be controlled and the light can be turned on or off with millisecond precision via the microcontroller. We attached LEDs of three colors to the DIRI illuminator for WSI. Before use, the WSI system was calibrated using the VS-ASW software and the recommended calibration slide from the manufacturer. For measuring light intensity in the experiment, we used Illuminance Meter T-10 (Minolta, Tokyo, Japan).

## Results and Discussion

### Color balance adjustment

**Fig 4:**
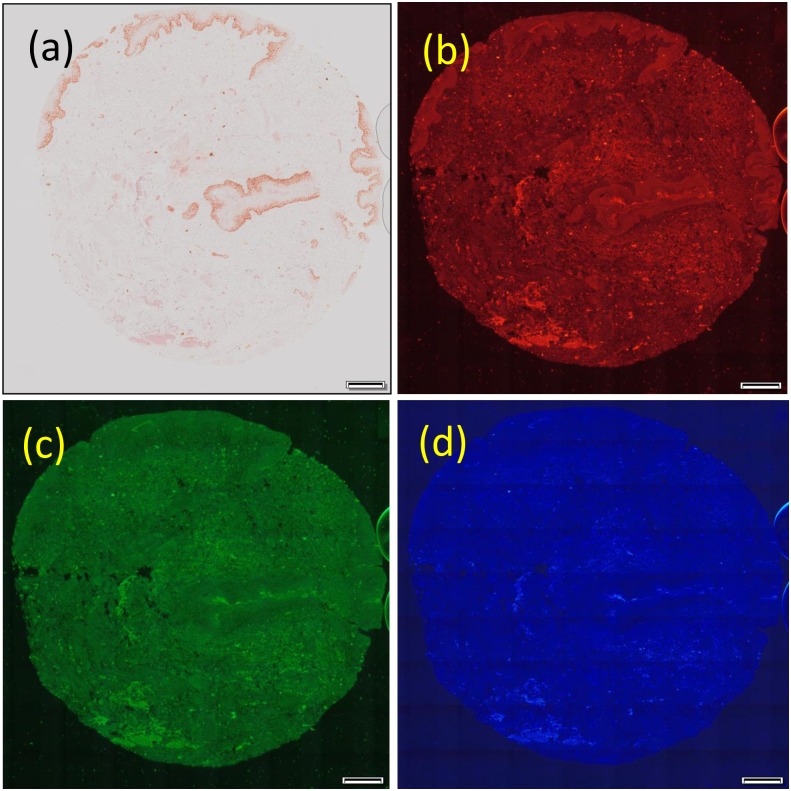
Brightfield image and red, green, and blue darkfield internal reflection illumination (DIRI) images of tissue microarray (TMA) sample. (a) Illuminated by brightfield, (b) red, (c) green, and (d) blue. All images were taken with UplanSapo 40× oil (NA 0.95) using the VC50 color camera. The exposure times are; (a) 8.7 ms, (b-d) 700 ms. The sample was skin tissue. Scale bars in the Figs represent 200 μm.

We observed a tissue microarray (TMA) sample of skin tissue using brightfield and color DIRI illumination. The color balance could be set on the color DIRI using a NeoPixel intensity interface where each color LED can be adjusted on a scale of intensity from 0 to 255, 255 being the maximum. Fig 4 shows the TMA sample illuminated by: (a) brightfield illumination with a halogen bulb; (b) DIRI illumination using red LEDs, with a maximum illumination intensity of 1800 lux and NeoPixel intensity of red 255; (c) DIRI illumination with green LEDs, with a maximum illumination intensity of 3700 lux and NeoPixel intensity of green 255; and (d) DIRI illumination with blue LEDs, with a maximum illumination intensity of 1000 lux and NeoPixel intensity of blue 255. The illumination intensity was measured using a Minolta intensity meter. We confirmed that DIRI with red, green, or blue LEDs properly illuminated the TMA sample with each color, and the DIRI image provided a more detailed structure than that with brightfield illumination with white light. Although the image quality of Fig 4d is not high, we believe this is due to the low sensitivity of the CCD to blue light and could be improved by another sensor.

**Fig 5:**
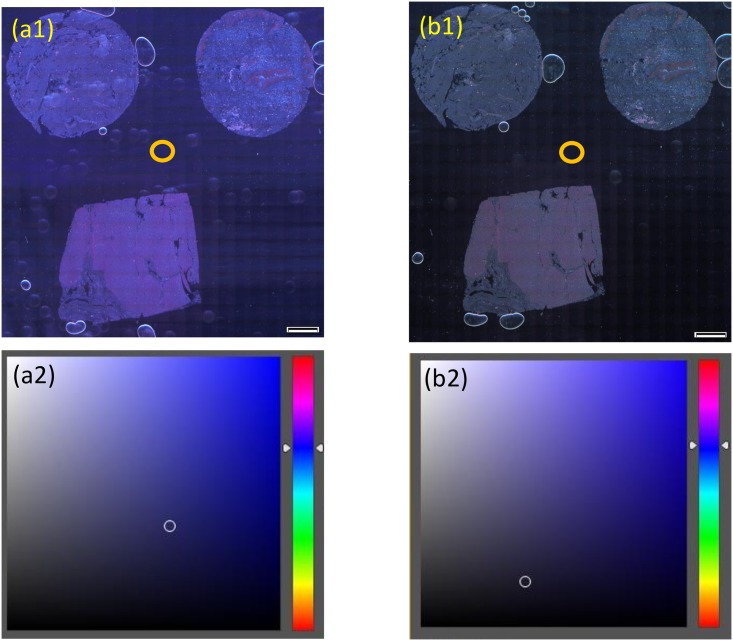
Maximum intensity illumination of color DIRI and color balance modification. (a1) Image taken with the maximum intensity of color DIRI with red 255, green 255, and blue 255. (a2) Photoshop color chart of a1 showing the location in the color balance. (b1) Image taken with the modified color DIRI with red 150, green 255, and blue 100. (b2) Photoshop color chart of b1 showing the location in the color balance. All images (a1, b1) were taken with UplanSapo 40× oil (NA 0.95) using the VC50 color camera. The exposure time is 400 ms. The sample was skin tissue. Scale bars in the Figs represent 200 μm.

Fig 5 shows the advantages of the color balancing properties of color DIRI. We observed TMA samples of three different tissues: brain tissue (top left), skin tissue (top right), and liver tissue (bottom), with color DIRI. The first set (Fig 5a1) used a maximum illumination intensity of 6400 lux. The NeoPixel intensity set-up was red 255, green 255, and blue 255. Our sensory evaluation results showed that the background of the image was blue in color. To improve the color tone, we modified the NeoPixel intensity to red 150, green 255, and blue 100. The resulting illumination intensity was 5000 lux. The resultant image is shown in Fig 5b1. Our sensory evaluation results showed that the background of the image of b1 had less blue color than a1.

Color maps measured by Photoshop software at the center of Fig 5a1 and 5b1 are shown in Fig 5a2 and 5b2, respectively. The background color can be analyzed in terms of hue (H), saturation (S), and brightness (B). Image a1 had the values of H: 241, S: 60, and B: 38, whereas image b1 had H: 247, S: 40, and B: 17. By reducing the intensities of red and blue light, the background color became a more natural black in b1. We see that the background color in b1 is less blue and darker than that in a1.

These results clearly illustrate that the independent control of the three-color LEDs could improve the color balance of DIRI illumination compared with white-LED illumination. We think that the color balance function of our color DIRI is novel and advantageous compared with the standard Hausmann’s darkfield and white DIRI. Comparisons to Hausmann’s darkfield and white DIRI will be presented later.

### Observation of DAB-stained brain slice

**Fig 6:**
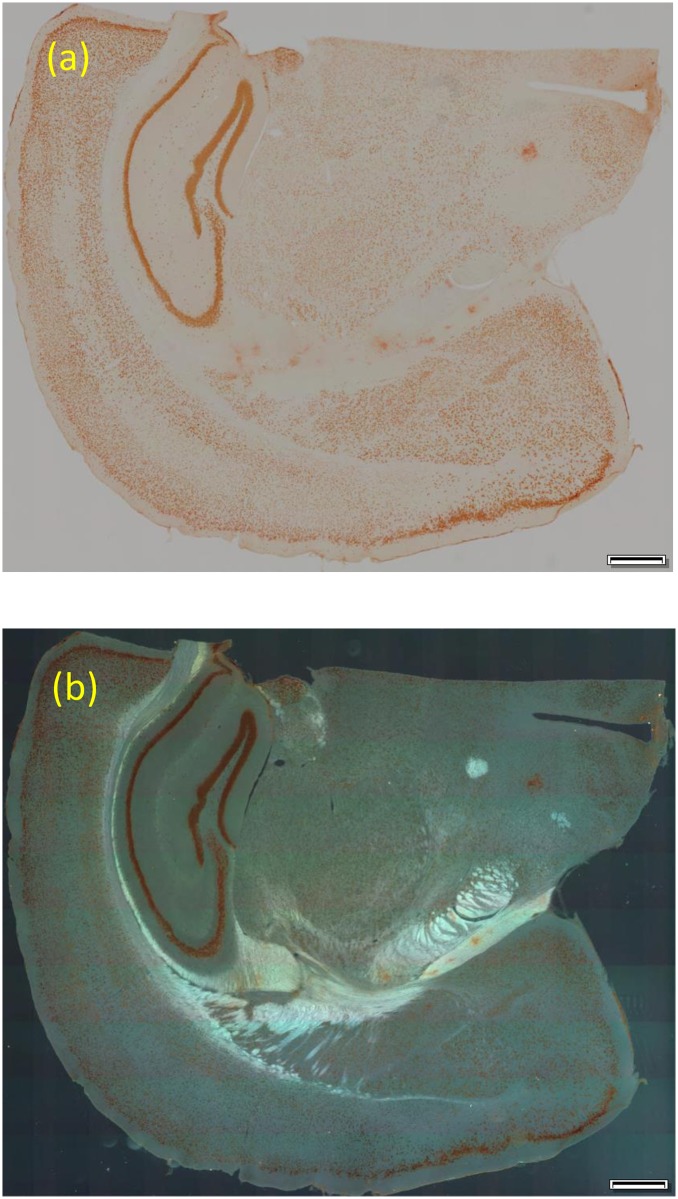
Diaminobenzidine (DAB)-stained brain slices. (a) Brightfield image. (b) Color DIRI image with red 150, green 225, and blue 100. Images were taken with PlanApon 2× (NA 0.08) (a), and UPlanSapo20× (NA 0.75) (b). The exposure times are (a) 4 ms, and (b) 150 ms. Both are using the VC50 color camera. Scale bars indicate 0.5 mm.

3,3′-Diaminobenzidine (DAB) staining is one of the most commonly used staining methods. We thus checked the utility of our color DIRI using a DAB-stained brain tissue section. Fig 6a shows the sample under brightfield illumination, while Fig 6b shows the sample observed by using color DIRI. The light intensity was set as red 150, green 225, and blue 100, for a total of 5,000 lux. The DIRI image shows the structure of the corpus callosum, whereas the brightfield image does not. The results indicate that color DIRI is useful for observing the structures of DAB-stained samples.

If two illuminators (such as transmitted and DIRI) are used, the color balance of one of them has to be adjusted. Otherwise, it is necessary to change the color balance of the camera itself when switching the illuminator. We included the ability to change the color balance in the darkfield illuminator (color DIRI); thus, it is possible to switch to a brightfield mode without changing the camera settings. This is ideal for automated image acquisition.

### Observation of brain slice without visible color staining

**Fig 7:**
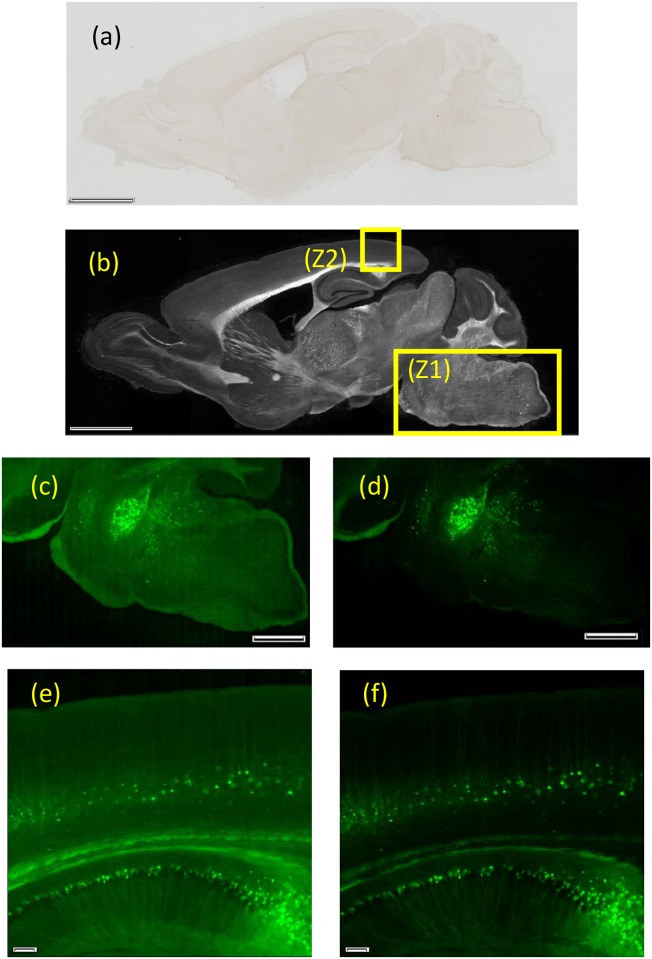
Brain slice images obtained by color DIRI. (a) Brightfield image taken with PlanApon 2× (NA 0.08) using the VC50 color camera. The exposure time is 4.6 ms. (b) Darkfield image taken with DIRI with Orca R2 B/W camera. The exposure time is 100 ms. (c) LED-illuminated fluorescence image of area Z1 taken with UPlanSapo40× (NA 0.95) with Orca R2 B/W camera. The exposure time is 100 ms. (d) Epi-fluorescence image of area Z1 taken with UPlanSapo40× (NA 0.95) with Orca R2 B/W camera. The exposure time is 100 ms. (e) LED-illuminated fluorescence image of area Z2 taken with the UPLSAPO 40× silicon immersion lens (NA 1.25) with Orca R2 B/W camera. The exposure time is 100 ms. (f) Epi-fluorescence image of area Z2 taken with the UPLSAPO 40× silicon immersion lens (NA 1.25) with Orca R2 B/W camera. The exposure time is 100 ms. Scale bars in the Figs represent (a–d) 2 mm and (e, f) 0.1 mm.

We examined the effectiveness of color DIRI in the imaging of samples without visible color staining. Fig 7 shows images of a brain section not stained by a visible dye. We first obtained a large image of the brain slice using a PlanApon 2× objective lens with brightfield illumination (Fig 7a). We can see that the image contrast is low, making it difficult to distinguish structures in the sample. The illumination method was then switched to color DIRI using the same objective lens, as shown in Fig 7b. The color DIRI image has significantly higher contrast than the brightfield image, indicating the effectiveness of color DIRI, even for a sample without staining.

### Observation of brain slice staining with a fluorescent marker

First, we examined the fluorescence imaging of the brain using blue LED excitation illumination from the color DIRI system. We compared the epifluorescence image and blue LED excitation image using fluorescent proteins (YFP-H). Using the objective lens UplanSapo 40×, NA 0.9, we observed the area Z1, which is highlighted in Fig 7b.

Second, we acquired a standard epifluorescence image (Fig 7d). Moreover, by using an excitation filter, we obtained the median values of the excitation wavelength, full width at half maximum (FWHM), and full bandwidth to be 494, 20, and 25 nm, respectively. The illumination wavelength was approximately 481.5–506.5 nm. Because of a sharp and narrow cut-off bandpass filter, the full bandwidth was only 5 nm wider than FWHM. The background of the fluorescence image was dark, and the fluorescence image could be seen at a very high contrast.

In addition, in Fig 7c, the wavelength of the illuminated blue LED and the median of the excitation wavelength was 467 nm, the FWHM was 10 nm, and the full bandwidth was 100 nm. The illumination wavelength was approximately 417–517 nm. However, the full bandwidth was 90 nm wider than that of the FWHM as a broad band blue LED was used. Fluorescent proteins (YFP-H) were excited using the blue LED. From Fig 7c, we can analyze that the color DIRI can produce the fluorescent image. However, in the case of the fluorescence image in Fig 7c, the LED light with wavelength of approximately 517 nm was making bleed through of excitation filter (515–545 nm) and into camera. This broader wavelength of blue LED light, especially 517 nm light passed through the emission filter, which created a slightly brighter background in Fig 7c than that in Fig 7d. Although blue LED excitation provides a slightly brighter background, it is convenient for us to use the color DIRI in many practical situations as it allows us to easily adjust the wavelength of light during fluorescent imaging.

Another advantage of the color DIRI is that it provides easy and automatic image acquisition with oil objectives. As shown in Fig 7(e), we examined color DIRI together with a UPLSAPO40×, NA 1.25, silicone oil immersion objective lens. Easy and automatic image acquisition was successfully achieved in this study. Fig 7(f) shows an image using epifluorescent imaging for comparison. The contrast for the image background in Fig 7(f) is higher than that in Fig 7(e); however, the performance for the color DIRI is acceptable.

Theoretically, if we can set a bandpass filter between a white LED and a slide glass sample, controlling the illumination wavelength is possible. However, the space between the LED and the slide sample is limited. Thus, it is difficult to mechanically install the bandpass filter. As our former white DIRI system could not acquire fluorescent images, the proposed color DIRI system demonstrates significant progress regarding imaging capabilities.

### Observation of photo response of *Euglena gracilis*

Next, we examined whether the color DIRI could be utilized to investigate the photo response of cells using *Euglena gracilis*, which has been a subject of intense investigations [14–19]. In this experiment, we used two kinds of illumination: one for sample observation and the other for photo stimulation. The illumination for sample observation was transmitted illumination generated by a halogen lightbulb with an SC68 sharp cut-off filter; this filter only transmits light of a wavelength longer than 680 nm. The SC68 filter, which is a long pass filter from 680 nm, can reduce the photo response of *E gracilis*, given that *E gracilis* shows phototaxis to shorter wavelengths [16, 17, 19]. The illumination for photo stimulation was generated by the color DIRI.

**Fig 8:**
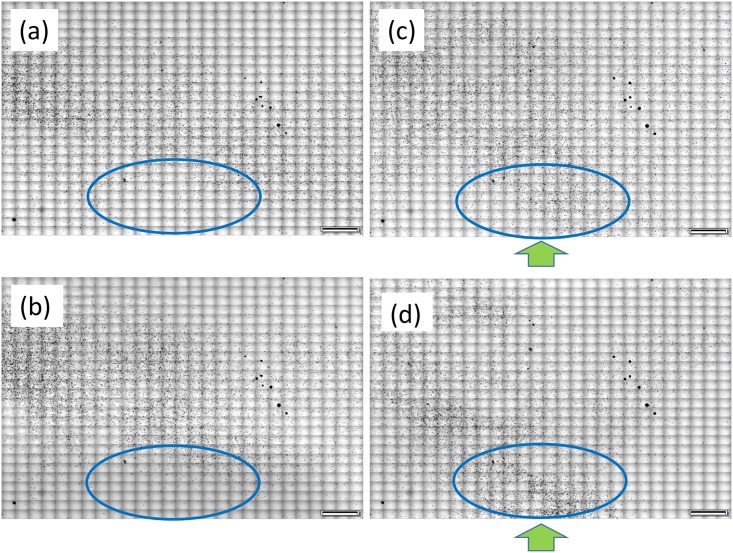
Photo responses of *E gracilis* cells stimulated by green LED. (a) Initial distribution of cells when the LED was off. Small black dots in the figure indicate cells. (b) The distribution of cells 10 min after loading when the LED was off. (c) The distribution of cells 30 min after turning on the green LED with an intensity of 5. Arrow indicates the position and direction of illumination of the LED. (d) Distribution of cells 10 min after increasing the light intensity to green 255. All images were taken with UPlanSapo10× (NA 0.4), and the scale bars in the figure represent 2 mm. All images were taken using Orca R2 color camera. The exposure time is 100 ms.

We placed *E gracilis* cells in a gap of 0.4 mm between a coverslip and a slide glass. When these cells were loaded, the DIRI LEDs were off. Initially, the cells were distributed across the entire region, as shown in Fig 8a. As a control, we acquired an image 10 min after the loading, as shown in Fig 8b. The cell distribution was similar to that in Fig 8a, and no accumulation of cells was observed near the LEDs, namely, in the blue ellipsoidal region.

Next, we turned one LED on, the intensity of which was set as follows: red 0, green 5, and blue 0. The light intensity was 4 lux. Fig 8c shows the image acquired 30 min after LED illumination. We can see that many cells had accumulated in front of the LED, as indicated by the blue ellipse. In other words, *E gracilis* had exhibited phototaxis, swimming towards the light. We then increased the light intensity to red 0, green 255, and blue 0 (740 lux). Fig 8d shows an image acquired 10 min after this increase. We can see that quite a large number of cells accumulated in front of the LED, which indicates that the cells showed a stronger photoresponse upon an increase of the light intensity. The color DIRI can illuminate cells with three different colors and from eight different positions. We thus believe that the present system is useful for investigating the photo response of various swimming microorganisms.

### Comparison with Hausmann’s darkfield and white LED DIRI

We compared three different darkfield illumination methods: Hausmann’s darkfield, white-LED DIRI, and three-color-LED DIRI. The results are summarized in Table 1. The top five lines in Table 1 describe the apparatus required for each method. Hausmann’s darkfield uses side illumination, which involves the direct connection of a lightbulb or fiber illumination. In the case of DIRI, however, as we previously explained, the same illumination set-up cannot be used because DIRI was developed for an automated microscope for WSI [7, 8]. DIRI should not be connected to a bulky tungsten or halogen lightbulb with a fiber bundle because the bulky units often interfere with the motorized stage. Given the small size of LEDs, they do not impose such a constraint.

**Table 1:** Comparison of Hausmann’s darkfield, white-LED DIRI, and three-color LED DIRI.

	Hausmann’s Darkfield	White LEDs DIRI	Three Color LEDs DIRI
**Apparatus**			
Compact design	N/A	Use	Use
Fiber bundle	Use	N/A	N/A
Halogen or tungsten Lamp	Use	N/A	N/A
White LED	N/A	Use	N/A
Three color LED	N/A	N/A	Use
**Functions**			
Illumination color balance correction	Use optical filter	N/A	Possible
Using excitation for fluorescence imaging	Use optical filter	N/A	Possible
Selected color and intensity change	Use optical filter	N/A	Possible
Selected illumination location	N/A	N/A	Possible
Photostimulator (controlling color, intensity and illumination location)	N/A	N/A	Possible

The bottom five lines in Table 1 explain the functions. When we take a color image with brightfield and darkfield modes, color adjustment is always required. Three-color DIRI has the ability to adjust the illumination color balance. This is essential for taking brightfield and darkfield images using the same camera, which was not possible using white-LED DIRI. In the case of color DIRI, we can take both images without changing the camera set-up.

As shown in Fig 7, fluorescent imaging can be performed only by color DIRI. This is one of its major advantages compared with the other two methods. Metal nanoparticles are also used in biological imaging. They exhibit a plasmonic resonance that induces unique optical properties. The excitation light for nanoparticles should have a shorter wavelength than the emission wavelength, which can be provided by color DIRI. Moreover, color DIRI can provide a darkfield image only using red illumination. The red illumination can prevent sample damage and photobleaching compared with white or blue light. Long-wavelength photons are less energetic and phototoxic. In addition, selective longer-wavelength darkfield illumination by color DIRI should reduce photobleaching and phototoxic effects. These characteristics of color DIRI are preferable for biological applications.

The color LEDs provide adjustable three-color illumination with eight sets of three-color LEDs. We can illuminate individual LEDs for selected locations. In addition, the LED controller has the ability to provide blinking illumination. These characteristics are favorable for investigating the photo responses of cells, as shown in Fig 8. Overall, the color DIRI significantly extends the abilities of WSI compared with the other two methods.

The color LEDs provide a photo stimulator function. This function indicates that the system can control the color, intensity, and area of the illumination. Meanwhile, a Hausmann’s illuminator, could not control the illumination location, even if an optical filter were to be added to it. Consequently, using Hausmann’s illuminator would not be appropriate for a photo stimulator.

### Color LED and LED array controller

The WS2812 array comprises three RGB LEDs, and we measured the distance between them. The distance between the blue and green LEDs was approximately 1.2 mm while the red LED was placed in between them. This fact indicates that one needs to carefully align the positions of the color LEDs relative to the slide glass. We also checked the commercially available microscope slide glass; the choices of available thickness were 0.8–1, 0.9–12, 1–1.2, and 1.2–1.5 mm. The most suitable of these was felt to be the 1.2–1.5 mm slide glass, as it can properly include the three RGB LEDs.

In this study, we used an Arduino UNO microcontroller to control the intensity of the LEDs. We only used a limited number of functions of the controller, such as the on/off switch and the illumination location selector. The use of some additional functions, such as the high-speed on/off switch and the select and switch illumination area, may be desirable in some situations. The update speed for Arduino UNO is 800 kHz, and each LED has a 24-bit color. Therefore, the update speed for eight LEDs is 1.25 ms. If the exposure time is on the order of this update speed, the illumination light makes frequency beating of the LED update speed against camera readout. More advanced features for controlling light are provided on the Arduino homepage “Fast, easy LED library for Arduino.” Moreover, another idea for improving frequency is provided by Tian and Waller [20]. If a better controller system such as Teensy 3.2 is used, a greater LED-array update speed can be obtained.

## Conclusions

We developed three-color DIRI and investigated its usefulness. This system offers the following advantages compared with previous darkfield illumination methods:

1. Color DIRI provides a function for color balance adjustment. The color balance of the resulting images can be controlled by pre-adjusting the DIRI color via a simple operation.
2. Color DIRI provides darkfield imaging with high-magnification objective lenses, such as 20× dry and 40× oil. Illuminating the DAB-stained mid-sagittal brain slice with color DIRI provided a clear image of the structure of the corpus callosum.
3. The blue DIRI provides fluorescence excitation. We clearly observed the fluorescence protein (YFP-H).
4. Color DIRI partially illuminates the sample with different colors. Potentially, less sample damage occurs with red DIRI illumination. The DIRI can also provide blinking illumination.
5. Color DIRI can be used as a photo stimulator for investigating the photo responses of cells.

These results clearly illustrate that color DIRI significantly extends the capability of WSI compared with the conventional methods.

## References

[1] Weinstein RS, Graham AR, Richter LC, Barker GP, Krupinski EA, Lopez AM, et al (2009) Overview of telepathology, virtual microscopy, and whole slide imaging: prospects for the future. *Hum Pathol* 40, 1057–1069. 10.1016/j.humpath.2009.04.006 19552937

[2] Pantanowitz L, Valenstein PN, Evans AJ, Kaplan KJ, Pfeifer JD, et al (2011) Review of the current state of whole slide imaging in pathology. *J Pathol Inform* 2, 36 10.4103/2153-3539.83746 21886892 PMC3162745

[3] Yagi Y, Gilbertson JR (2005) Digital imaging in pathology: the case for standardization. *J Telemed Telecare* 11, 109–116. 10.1258/1357633053688705 15901437

[4] Long RE, Smith A, Machotka SV, Chlipala E, Cann J, Knight B, et al (2013) Scientific and Regulatory Policy Committee (SRPC) paper: validation of digital pathology systems in the regulated nonclinical environment. *Toxicol Pathol* 41, 115–124. 10.1177/0192623312451162 22723045

[5] Higgins C (2015) Applications and challenges of digital pathology and whole slide imaging. *Biotech Histochem* 90, 341–347. 10.3109/10520295.2015.1044566 25978139

[6] Yagi Y (2011) Color standardization and optimization in whole slide imaging. *Diagn Pathol* 6 Suppl 1, S15, 21489185 10.1186/1746-1596-6-S1-S15 PMC3073208

[7] Kawano Y, Higgins C, Yamamoto Y, Nyhus J, Bernard A, Dong HW, et al (2013) Darkfield adapter for whole slide imaging: adapting a darkfield internal reflection illumination system to extend WSI applications. *PLoS One* 8, e58344, 10.1371/journal.pone.0058344 23520500 PMC3592912

[8] Kawano Y, Otsuka C, Sanzo J, Higgins C, Nirei T, Schilling T, et al (2015) Expanding imaging capabilities for microfluidics: applicability of darkfield internal reflection illumination (DIRI) to observations in microfluidics. PLoS One 10, e0116925 10.1371/journal.pone.0116925 25748425 PMC4352060

[9] Pluta M. (1989) Advanced Light Microscopy Volume 2 Specialized Methods. Elsevier 102–113.

[10] Miyawaki A, Homma H, Tamura HO, Matsui M, Mikoshiba K. (1996) Zonal distribution of sulfotransferase for phenol in olfactory sustentacular cells. *EMBO J* 15, 2050–2055 8641270 PMC450126

[11] Dong HW. Swanson LW. (2003) Projections from the rhomboid nucleus of the bed nuclei of the stria terminalis: implications for cerebral hemisphere regulation of ingestive behaviors. *J Comp Neurol* 463, 434–472. 10.1002/cne.10758 12836178

[12] Dong HW, Petrovich GD, Swanson LW. (2001) Topography of projections from amygdala to bed nuclei of the stria terminalis. *Brain Res Brain Res Rev* 38, 192–246. 11750933 10.1016/s0165-0173(01)00079-0

[13] Dong HW, Petrovich GD, Watts AG, Swanson LW. (2001) Basic organization of projections from the oval and fusiform nuclei of the bed nuclei of the stria terminalis in adult rat brain. *J Comp Neurol* 436, 430–455. 11447588 10.1002/cne.1079

[14] Jennings HS (1904) Contributions to the study of the behavior of lower organisms (No. 16). Carnegie institution of Washington.

[15] Lebert M, Hader DP (1999) Negative gravitactic behavior of Euglena gracilis can not be described by the mechanism of buoyancy-oriented upward swimming. *Adv Space Res* 24, 851–860. 11542631 10.1016/s0273-1177(99)00966-7

[16] Barghigiani C, Colombetti G, Franchini B, Lenci F (1979) Photobehavior of Euglena gracilis: action spectrum for the step-down photophobic response of individual cells. *Photochemistry and Photobiology* 29, 1015–1019.

[17] Iseki M, Matsunaga S, Murakami A, Ohno K, Watanabe M. (2002). A blue-light-activated adenylyl cyclase mediates photoavoidance in Euglena gracilis. Nature, 415(6875), 1047–1051. 10.1038/4151047a 11875575

[18] Tahedl H, Häder D.-P (2001). Automated biomonitoring using real time movement analysis of Euglena gracilis. *Ecotoxicology and Environmental Safety* 48, 161–169. 10.1006/eesa.2000.2004 11161690

[19] Ohashi M, Miyajima S, Ohi M (2012) Evaluation of the effects of the blinking cycle and duty ratio of red and blue light emitting diodes on the photosynthetic rate of Euglena. *Eco-Engineering*, 24(2): 43–49.

[20] Tian L, Waller L (2015) Quantitative differential phase contrast imaging in an LED array microscope. Optics express, 23(9), 11394–11403. 10.1364/OE.23.011394 25969234

